# Association of a Polymorphism in the *BIRC6* Gene with Pseudoexfoliative Glaucoma

**DOI:** 10.1371/journal.pone.0105023

**Published:** 2014-08-13

**Authors:** Humaira Ayub, Shazia Micheal, Farah Akhtar, Muhammad Imran Khan, Shaheena Bashir, Nadia K. Waheed, Mahmood Ali, Frederieke E. Schoenmaker-Koller, Sobia Shafique, Raheel Qamar, Anneke I. den Hollander

**Affiliations:** 1 Department of Biosciences, COMSATS Institute of Information Technology, Islamabad, Pakistan; 2 Department of Ophthalmology, Radboud University Nijmegen Medical Centre, Nijmegen, The Netherlands; 3 Al-Shifa Trust Eye Hospital, Rawalpindi, Pakistan; 4 Department of Human Genetics, Radboud University Nijmegen Medical Centre, Nijmegen, The Netherlands; 5 Department of Ophthalmology, Tufts University School of Medicine, Boston, Massachusetts, United States of America; 6 Al-Nafees Medical College & Hospital, Isra University, Islamabad, Pakistan; Sudbury Regional Hospital, Canada

## Abstract

Recently an association was observed between alleles in genes of the unfolded protein response pathway and primary open angle glaucoma (POAG). The goal of the current study is to investigate the role of these two genes, protein disulphide isomerase A member 5 (*PDIA5*) and baculoviral IAP repeat containing 6 (*BIRC6*), in different forms of glaucoma. 278 patients with POAG, 132 patients with primary angle closure glaucoma (PACG) and 135 patients with pseudoexfoliative glaucoma (PEXG) were genotyped for single nucleotide polymorphisms (SNPs) rs11720822 in *PDIA5* and 471 POAG, 184 PACG and 218 PEXG patients were genotyped for rs2754511 in *BIRC6*. Genotyping was done by allelic discrimination PCR, and genotype and allele frequencies were calculated. Logistic regression analyses were performed using R software to determine the association of these SNPs with glaucoma. The allele and genotype frequencies of rs11720822 in *PDIA5* were not associated with POAG, PACG or PEXG. The TT genotype of rs2754511 in *BIRC6* was found to be protective for PEXG (p = 0.05, OR 0.42 [0.22–0.81]) in the Pakistani population, but not for POAG or PACG. This study did not confirm a previously reported association of risk alleles in *PDIA5* and *BIRC6* with POAG, but did demonstrate a protective role of the T allele of rs2754511 in the *BIRC6* gene in PEXG. This supports a role for the unfolded protein response pathway and regulation of apoptotic cell death in the pathogenesis of PEXG.

## Introduction

Glaucoma is an optic neurodegenerative disorder [1] that leads to gradual loss of vision due to degeneration of the retinal ganglion cells [2]. Two subtypes of primary glaucoma can be distinguished, primary open angle glaucoma (POAG) and primary angle closure glaucoma (PACG), which are associated with different anatomical defects [3]. The most common form of secondary glaucoma is pseudoexfoliative glaucoma (PEXG), which is generally characterized by the appearance of dandruff-like grayish white flakes deposited over the iris, lens, and ciliary epithelium. This material collecting in the anterior angle has been related to raised intra ocular pressure (IOP) and ultimately glaucoma [4]–[6].

With advancing age, the exposure of the eye to various stress-inducing factors increases, which can damage the integrity of the trabecular meshwork. These majorly include free radicals, reactive oxygen species (ROS) [7], [8] and protein aggregation [9], which elevate oxidative stress in the eye. In PEXG and POAG the damage due to oxidative stress [8] can also succumb into mitochondrial damage and neuronal death of retinal ganglion cells eventually leading to vision loss [10]–[12].

A previous genome-wide analysis in a *Drosophila* ocular hypertension model identified transcripts with altered regulation, and showed induction of the unfolded protein response (UPR) upon overexpression of transgenic human glaucoma-associated myocilin [13], [14]. Single nucleotide polymorphisms (SNPs) in two genes involved in reduction of endoplasmic reticulum (ER) stress, protein disulphide isomerase family A member 5 (*PDIA5*) and Baculoviral inhibitor of apoptosis repeat-containing 6 (*BIRC6*), have been recently shown to be significantly associated with POAG [15], [16]. Since no other studies have yet attempted to replicate this finding, we investigated both SNPs in a cohort of POAG patients from Pakistan. In addition, we extended the analysis to patients with PACG and PEXG, as recent studies have suggested that common genetic factors might contribute to various forms of glaucoma [17].The aim of this study was to investigate the role of rs11720822 in *PDIA5* and rs2754511 in *BIRC6* in POAG, PACG and PEXG patients from the Pakistani population.

## Methods

### Ethics statement

This study has been approved by the Department of Biosciences, Ethics Review Committee and conforms to all of the norms of the Helsinki Declaration. Written informed consent was obtained from all participants.

### Patients and control individuals

In the present study 471 POAG, 184 PACG and 218 PEXG patients were recruited from different ophthalmological centers in Pakistan. The diagnosis of POAG and PACG was made as described previously [18]. PEXG was diagnosed on the basis of clinical history, cup-to-disc ratio (CDR) and intraocular pressure (IOP) measurements. In order to detect the exfoliative deposits on the pupillary border and the iris, slit lamp biomicroscopy was initially performed without dilation of the pupil, and subsequently after pupil dilation the patients were re-examined to detect the presence of white deposits on the anterior lens surface. Angles were measured with gonioscopy to discriminate between narrow and open angles. 160 unaffected controls, belonging to the same ethnic background as the patients, were classified on the basis of absence of any exfoliate deposits, normal gonioscopic observations and normal CDR and IOP values. The processing and DNA isolation from whole blood was performed as described previously [18].

### Genotyping

Two intronic SNPs, rs11720822 in *PDIA5* and rs2754511 in *BIRC6*, were genotyped in the POAG, PACG, PEXG patients and control individuals using Taqman allelic discrimination assays performed by Real-time PCR (Applied Biosystems 7900HT Fast System and Sequence Detection Systems Software v2.3, Foster City, CA). The polymerase chain reaction (PCR) amplification was performed according to the protocol of the manufacturer using 10 ng of DNA in a reaction mixture of 4 µl. After an initial denaturation step of 12 minutes at 95°C, 50 cycles of amplification were performed for 15 seconds at 92°C and termination for 90 seconds at 60°C.

### Statistical Analysis

Allele frequencies between the unaffected controls and the three patient groups (POAG, PACG and PEXG) were compared with the Pearson χ2 test using online free link available (http://statpages.org/ctab2x2.html). The Genotype frequencies were calculated by logistic regression analysis keeping age and gender as covariates. The data was statistically analyzed by using R software (R Core Team (2012). R: A language and environment for statistical computing. R Foundation for Statistical Computing, Vienna, Austria. ISBN 3-900051-07-0, URL http://www.R-project.org/). Moreover Bonferoni correction was applied to the individual p-values generated from logistic regression analysis.

## Results and Discussion

Patients and controls included in the current study were age and gender matched (table 1). The mean age (± standard deviation) of the controls was 48.1±13.2 years, of patients with POAG 48.3±16.5 years, PACG 45.5.±16.5 years and PEXG 50.09±14.1 years. In total 321 healthy subjects (52% males and 48% females), 471 patients with POAG (51% males and 49% females), and 184 patients with PACG patients (49.5% males and50.5% females) and 218 PEXG (48% males and 52% females) were enrolled in the study. The majority of the patients was treated with medications such as β-blockers, or underwent surgery (trabeculectomy) to lower IOP.

**Table 1:** Demographic and clinical features of the control, POAG, PACG and PEXG cohorts.

	Controls	POAG	p-value	PACG	p-value	PEXG	p-value
Age	48.1±13.2	48.3±16.5	>0.05	45.5.±16.5	>0.05	50.09±14.1	>0.05
Gender							
Males	52%	51%	>0.05	49.5%	>0.05	48%	>0.05
Females	48%	49%		50.5%		52%	
IOP	16.4±2.3	30.5±11.6	<0.05	26.4±10.2	<0.05	28.4±10.5	<0.05
CDR	0.39±0.18	0.85±.63	<0.05	0.67±.27	<0.05	0.76±.23	<0.05

The allele and genotype frequencies of rs11720822 in *PDIA5* were not significantly different between POAG, PACG and PEXG compared to control individuals (Table 2). The T allele of rs2754511 in *BIRC6* was found on 35% of control alleles, while it was found on 32% of POAG alleles, 35% of PACG alleles, and 28% of PEXG alleles. The frequency of the T allele is significantly lower in PEXG patients as compared to controls (p = 0.01; OR 0.72 [95% CI 0.55–0.94]), suggesting it has a protective effect (Table 2), however this association did not remain significant when Bonferoni correction was applied to the data (p = 0.06).

**Table 2:** Results of logistic regression analysis for Genotype and allele frequency of SNPs rs11720822 in *PDIA5* and rs2754511 in *BIRC6* in POAG, PCAG and PEXG patients and control individuals.

*PDIA5* rs11720822	POAG Est.	Z value	OR(95%CI)	P-value/p^b^	PACG Est.	Z value	OR(95%CI)	p-value/p^b^	PEXG Est.	Z value	OR(95%CI)	p-value/p^b^
**CC**	0.07	0.16	0.9 (0.37–2.26)^a^	0.86/1.00^b^	0.22	−0.45	0.79 (0.29–2.13)^a^	0.64/1.00^b^	0.60	1.30	1.82(0.74–4.48)^a^	0.18/1.00^b^
**CT**	17.90	0.02	5.95(0.00-Inf)^a^	0.98/1.00^b^	12.89	0.01	3.69 (0.00-Inf)^a^	0.98/1.00^b^	15.55	0.01	5.68(0.00-Inf)^a^	0.98/1.00^b^
**TT**												
**Allele Frequency**												
**C**	-	-	0.74 (0.41–1.34)^c^	0.28/1.00^b^	-	-	1.00 (0.51–1.96)^c^	0.99/1.00^b^	-	-	1.15 (0.60–2.19)^c^	0.66/1.00^b^
**T**	-	-			-	-			-	-		
***BIRC6*** rs2754511												
**AA**	0.39	−2.53	0.67 (0.49–0.91)^a^	0.01/0.06^b^	0.05	−0.28	0.94 (0.62–1.41)^a^	0.77/1.00	0.35	−1.87	0.70 (0.48–1.01)^a^ 0.42	0.06/0.36^b^
**AT**	0.18	0.81	0.83 (0.52–1.30)^a^	0.41/1.00^b^	0.18	0.59	1.20 (0.65–2.22)^a^	0.54/1.00^b^	0.84	2.58	(0.22–0.81)^a^	0.009/0.05^b^
**TT**												
**Allele Frequency**												
**A**	-	-	0.85 (0.68–1.06)^c^	0.15/0.90^b^	-	-	0.95(0.72–1.26)^c^	0.28/1.00^b^	-	-	0.72 (0.55–0.94)^c^	0.01/0.06^b^
**T**	-	-			-	-			-	-		

a Age and gender adjusted OR and (95%CI) from multivariate logistic regression analysis; p^b^Bonferoni corrected p values; ^c^OR and (95%CI) from univariate logistic regression analysis; Est., estimates

“Logistic regression analysis was conducted to adjust for age and gender which showed that the homozygous TT genotype of the studied *BIRC6* polymorphism is protective for PEXG (p = 0.009), which remained significant even after the Bonferoni correction (p = 0.05).”

Addition of 163 controls, 194 POAG cases, 47 PACG cases and 88 PEXG cases to the previous data improved the statistical power and supported the previous association (p = 0.03) of PEXG with the BIRC6 SNP rs2754511 (p = 0.01). Moreover the application of logistic regression analysis further strengthened the results (p = 0.009).

The two SNPs in *PDIA5* and *BIRC6* were previously associated with POAG in the Salt Lake City and San Diego populations [16]. Since recent studies have suggested that common genetic factors might contribute to various forms of glaucoma, we extended our study to PACG and PEXG. We did not find an association of these SNPs with either POAG or PACG in our population, nor did we find an association of the *PDIA5* gene polymorphism with PEXG. According to the dbSNP database, occurrence of the T allele of rs11720822 in *PDIA5* is very rare in various populations of the world including the Asian and African populations (http://www.ncbi.nlm.nih.gov/projects/SNP/snp_ref.cgi?rs=rs11720822), which supports our finding, as we also do not see high occurrence of the allele in our population. Our study did detect a moderate association of the *BIRC6* rs2754511 polymorphism with PEXG in the Pakistani population (p = 0.05). In agreement with the previous findings in the Salt Lake City and San Diego populations, demonstrating a protective effect of the T allele of rs2754511 on the development of POAG [16], this allele in homozygous form (TT) was also found to be protective for PEXG in our study. In this study, we investigated only the SNPs that were significantly associated with POAG in both the San Diego and Salt Lake City cohorts. We therefore cannot exclude that other SNPs in *BIRC6* or *PDIA5* may be associated with glaucoma in the Pakistani population.

BIRC6 is ubiquitin carrier protein involved in the protection of the cell against apoptosis and reduces cellular stress [16], [19]. Increased intraocular pressure, ROS and free radicals create a stressful environment in the eye [8], [20]. In the ER, stress can be accompanied by the aggregation of misfolded proteins. The accumulation of misfolded proteins can activate a cytoprotective signal response known as unfolded protein response (UPR), which triggers the activator functions like adaptation, alarm and apoptosis [12]. When stress is prolonged and adaptation and alarm fail to pull the cell back to normal condition, the UPR results in activation of apoptosis [21] and also elicits an inflammatory response in order to restore the normal environment of the cell. This mechanism has been found to be involved in the pathogenesis of many neurodegenerative disorders like Alzheimer's disease, Parkinson's disease and cerebral ischemic insults [22].

In PEXG and POAG the damage due to oxidative stress can succumb into mitochondrial damage [8], [10], [11]. The extracellular matrix of the trabecular meshwork is disrupted as a consequence of damage to the mitochondria, a characteristic mechanism involved in the pathogenicity of POAG and PEXG [8]. Konstas *et al*. [23] have observed excessive mitochondrial alterations in PEXG. The highest level of mitochondrial damage and mitochondrial loss per cell was seen in PEXG as compared to POAG, which justifies its more aggressive nature.

Zenkel *et al* have reported differential expression of ECM proteins and stress response genes in eyes of PEXG patients compared to eyes of normal healthy controls [24]. The expression of ECM genes is upregulated, resulting in aggregation of ECM proteins. Glutathione S-transferase 1, which is involved in protection from oxidative stress, is downregulated [24]. In addition, clusterin, an efficient extracellular chaperone, is downregulated in PEXG eyes, resulting in aggregation of pathologic ECM proteins [25]. Consequently, abnormal proteins accumulate, resulting in the formation of pseudoexfoliative material [25]. In the anterior chamber this hinders the outflow of aqueous humor by clogging the trabecular meshwork, which results in elevation of the IOP [26], [27]. All these stresses succumb in severe degenerative changes in PEXG.

Apoptosis might be one of the various mechanisms that is involved in the degeneration of retinal ganglion cells in PEXG. BIRC6 is an anti-apoptotic protein, which promotes cell survival by inhibiting caspases [28]. Downregulation of BIRC6 by various polymorphisms and mutations leads to upregulation of p53, resulting in mitochondrial-mediated apoptotic cell death [29]. As a consequence of stress, cytochrome C is released from mitochondria, which activates caspases and thus resulting in the degeneration and death of the cells [30].

Recent GWAS studies by Nakano *et al*., for the Japanese population [31], Gibson *et al*., for British population [32], Ramdas *et al*., Dutch population [33] have shown few loci and SNPs to be associated with Glaucoma but these studies did not find any association of the BIRC6 gene polymorphism rs2754511 with the POAG cohort. It was seen that the BIRC6 SNP was not covered in the maps used in those studies therefore that might be the reason of not finding rs275411 association. Contrary to the studies mentioned above we and Carbone et al found significant association of the SNP with PEXG and POAG respectively. As SNPs have individual, ethnicity and population specific role in disease etiology, therefore the association that we found could be due to divergent background of the studied populations [34].

In conclusion, our study demonstrates that the T allele of the rs2754511 in the *BIRC6* gene plays a protective role in PEXG patients of the Pakistani population. This supports a role for the UPR pathway and regulation of apoptotic cell death in the pathogenesis of PEXG.
